# Molecular alterations associated with liver metastases development in colorectal cancer patients

**DOI:** 10.1038/bjc.2011.184

**Published:** 2011-06-14

**Authors:** S C Bruin, Y He, I Mikolajewska-Hanclich, G-J Liefers, C Klijn, A Vincent, V J Verwaal, K A de Groot, H Morreau, M-L F van Velthuysen, R A E M Tollenaar, L J van ‘t Veer

**Affiliations:** 1 Department of Surgery, Division of Experimental Therapy, The Netherlands Cancer Institute – Antoni van Leeuwenhoek Hospital, Plesmanlaan 121, 1066 CX Amsterdam, The Netherlands; 2 Division of Experimental Therapy, The Netherlands Cancer Institute – Antoni van Leeuwenhoek Hospital, Plesmanlaan 121, 1066 CX Amsterdam, The Netherlands; 3 Department of Surgery, Leiden University Medical Center, Albinusdreef 2, 2333 ZA Leiden, The Netherlands; 4 Department of Molecular Biology, The Netherlands Cancer Institute – Antoni van Leeuwenhoek Hospital, Plesmanlaan 121, 1066 CX Amsterdam, The Netherlands; 5 Delft Bioinformatics Lab., Delft University of Technology, PO Box 5031, 2600 GA Delft, The Netherlands; 6 Department of Biometrics, The Netherlands Cancer Institute – Antoni van Leeuwenhoek Hospital, Plesmanlaan 121, 1066 CX Amsterdam, The Netherlands; 7 MRC-Holland, Willem Schoutenstraat 6, 1057 DN Amsterdam, The Netherlands; 8 Department of Pathology, Leiden University Medical Center, Albinusdreef 2, 2333 ZA Leiden, The Netherlands; 9 Department of Pathology, The Netherlands Cancer Institute – Antoni van Leeuwenhoek, Plesmanlaan 121, 1066 CX Amsterdam, The Netherlands

**Keywords:** colorectal cancer, liver metastases, chromosome 20q, PI(3)K signalling pathway, *PIK3CA*, *KRAS*

## Abstract

**Background:**

Understanding the molecular biology of colorectal cancer (CRC) provides opportunities for effective personalised patient management. We evaluated whether chromosomal aberrations, mutations in the PI(3)K signalling pathway and the CpG-island methylator phenotype (CIMP) in primary colorectal tumours can predict liver metastases.

**Methods:**

Formalin-fixed paraffin-embedded material from primary colorectal tumours of three different groups were investigated: patients with CRC without metastases (M0, *n*=39), patients who were treated with hyperthermal intraperitoneal chemotherapy for CRC metastases confined to the peritoneum (PM, *n*=46) and those who had isolated hepatic perfusion for CRC metastases confined to the liver (LM, *n*=48).

**Results:**

All samples were analysed for DNA copy number changes, *PIK3CA*, *KRAS*, *BRAF* mutations, *CIMP* and microsatellite instability. The primary CRCs of the LM group had significantly higher frequency of amplified chromosome 20q (*P*=0.003), significantly fewer mutations in the PI(3)K signalling pathway (*P*=0.003) and fewer CIMP high tumours (*P*=0.05). There was a strong inverse correlation between 20q and the PI(3)K pathway mutations.

**Conclusion:**

The development of CRC liver metastases is associated with amplification of chromosome 20q and not driven by mutations in the PI(3)K signalling pathway.

Colorectal cancer (CRC) is the second leading cause of cancer death in the Western world. The WHO estimates that 945 000 new cases occur yearly, with 492 000 deaths (Weitz *et al*, 2005). The overall 5-year survival is 57%, and up to 50% of patients will develop metastases. Distant metastases are responsible for the great majority of CRC deaths, mainly due to liver metastases (Welch and Donaldson, 1979; Galandiuk *et al*, 1992; Sadahiro *et al*, 2003). Of all patients who die of advanced CRC, 60–70% have liver metastases (Weiss *et al*, 1986). Even with the use of targeted drugs, the overall survival in patients with non-resectable CRC liver metastases is only 2 years, and late detection of liver metastases is still fatal. Hepatic resection is the only potentially curative treatment for a subset of patients with colorectal liver metastases. In these patients with resectable liver metastases, a 5-year survival of 30% can be achieved, and up to 20% of this population will still be alive after 10 years. The eligibility for hepatic surgery depends on whether all metastases are resectable and an adequate liver function can be maintained. There should be no extrahepatic disease, with the possible exception of few resectable lung metastases (Rees *et al*, 2008). Fong *et al* (1999) created a clinical risk score to identify patients who would most benefit from liver resection and showed that two of the five factors were determined by the number and size of the metastases in the liver. Therefore, early detection of liver metastases is of utmost priority and can result in more radical surgery and thus long-term survival (Rodriguez-Moranta *et al*, 2006).

The current tumour node metastases staging system of CRC has great clinical utility for prognosis and adjuvant treatment decision, but provides no information about a site-specific risk for liver metastases. A better understanding of the molecular biology of CRC progression has the potential to provide this information.

Colorectal cancer arises and processes through an adenoma–carcinoma sequence. This adenoma–carcinoma sequence follows well-defined steps of histological stages, each characterised by distinct mutations in oncogenes and tumour suppressor genes (Fearon and Vogelstein, 1990; Lengauer *et al*, 1997). The majority (85%) of CRC are chromosomal instable, characterised by allelic losses, chromosomal amplifications, translocations, as well as gene mutations (Lengauer *et al*, 1997; Thiagalingam *et al*, 2001; Jass *et al*, 2002; Jass, 2007).

One of the pathways often affected in CRC is the PI(3)K signalling pathway. This pathway has a central role in tumorigenesis by regulating cell growth, differentiation and apoptosis (Vivanco and Sawyers, 2002; Samuels *et al*, 2004; Shaw and Cantley, 2006). In this pathway, *PIK3CA*, *KRAS* and *BRAF* genes are frequently activated by mutations in various tumour types, including CRC (Samuels *et al*, 2004; Oikonomou and Pintzas, 2006; Cardoso *et al*, 2007; Kato *et al*, 2007) with frequencies of 10–30% (Samuels *et al*, 2004; Velho *et al*, 2005; Kato *et al*, 2007; Barault *et al*, 2008; Nosho *et al*, 2008; Ogino *et al*, 2009b), 30–40% (Vogelstein *et al*, 1988; Andreyev *et al*, 1998; Cardoso *et al*, 2007) and 5–22% (Yuen *et al*, 2002; Cardoso *et al*, 2007), respectively. Mutations in any one of these three genes will activate the PI(3)K signalling pathway and increases the transcription of different oncogenes, such as *C-MYC*, *CREB*, *NF-kB* and others (Barault *et al*, 2008), resulting in unrestricted cell growth.

There is a potential role of this signalling pathway in predicting survival (Kato *et al*, 2007; Barault *et al*, 2008); however, little is known about the relation between mutations in primary colorectal tumours and the risk of developing liver metastases. Furthermore, in our recent work we showed that an amplification of chromosome 20q is strongly related with the development of liver metastases (Bruin *et al*, 2010).

Here, we evaluated whether chromosomal aberrations and/or mutations in the PI(3)K signalling pathway and CpG-island methylator phenotype (CIMP) in primary colorectal tumours can predict liver metastases. Improved understanding of the biological mechanisms underlying liver metastases may allow tailored follow-up and potentially adapted management in patients with early-stage CRC.

## Materials and methods

### Patients and tumour samples

Formalin-fixed paraffin-embedded material (FFPE) from primary colorectal tumours of three different groups were investigated: patients with CRC who had not developed metastases, median follow-up 8 years (M0, *n*=39); patients who had been treated with hyperthermal intraperitoneal chemotherapy for synchronous or metachronous CRC metastases confined to the peritoneum (PM, *n*=46) (Verwaal *et al*, 2003, 2008); and patients who had been treated with isolated hepatic perfusion for CRC synchronous or metachronous metastases confined to the liver (LM, *n*=48) (van Iersel *et al*, 2008). These three specific groups were selected to identify specific markers for LM, which are not particular for other metastatic localisations such as PM, nor characteristic for CRC that do not metastasise. The LM and PM groups were carefully screened at the time of diagnosis to ensure they were free of other distant metastases (Marinelli *et al*, 1998; Verwaal *et al*, 2003). Clinical and pathological characteristics were retrieved from computerised medical registries and existing study databases (Marinelli *et al*, 1998; Verwaal *et al*, 2003; van Iersel *et al*, 2008).

We used anonymised tissue material from patients with consent following standard operational procedures in the respective hospitals, approved by the Institutional Review Board. Tissue handling was anonymised following the Declaration of Helsinki.

### DNA extraction and mutation analysis

DNA isolation was performed as described earlier (Joosse *et al*, 2007). Briefly, genomic DNA was isolated by proteinase K digestion after deparaffination from 10 × 10 *μ*m^2^ FFPE tissue sections containing at least 70% tumour cells from both the M0 and PM group. For the LM group, DNA was isolated from FFPE tissue block punches. These punches were taken out of the tissue blocks in the area with at least 60% tumour cells.

For the mutation analysis, exons 9 and 20 of *PIK3CA*, exon 1 of *KRAS* and exon 15 of *BRAF* were amplified by PCR and the presence of mutations was detected by direct sequencing using the BigDye Terminator Cycle Sequencing Kit (Applied Biosystems, Carlsbad, CA, USA). For all PCR products with sequence variants, both forward and reverse sequence reactions were repeated for confirmation. Primers used for the amplification and the PCR conditions are available upon request.

### Microsatellite instability

Analysis of microsatellite instability (MSI) was performed by using PCR and subsequent fragment analysis on an automated capillary sequence machine (ABI, 3730). The following eight markers were evaluated, including two mononucleotide markers (BAT 25 and BAT 40) and six dinucleotide markers (D1S158, D2S123, D5S346, D9S63, D17S250, D18S58). A tumour was considered to be MSI-high when three or more markers showed instability, MSI-low when one or two markers showed instability and MSI-stable when none of the eight markers showed instability (Kets *et al*, 2008).

### CIMP status

CpG-island methylator phenotype status was analysed by using the methylation-specific multiplex ligation-dependent probe amplification (MS-MLPA) kit (MRC-Holland, Amsterdam, the Netherlands; see Nygren *et al* (2005)). This MS-MLPA kit can be used to detect aberrant methylation of eight CIMP-specific promoters for CRC (CACNA1G, CDKN2A (p16), CRABP1, IGF2, MLH1, NEUROG1, RUNX3 and SOCS1) (Weisenberger *et al*, 2006; Ogino *et al*, 2009a). CpG-island methylator phenotype analysis was performed according to the manufacturer's protocol (SALSA MLPA kit, ME042-A1 CIMP; MRC-Holland). CpG-island methylator phenotype-high was defined as 6–8 methylated markers using the eight-marker CIMP panel and CIMP-low as 0–5 methylated markers (Ogino *et al*, 2007, 2009a).

### Chromosomal aberrations

DNA copy number changes were investigated using the 3.5k bacterial artificial chromosomes (BAC) array performing array comparative genomic hybridisation as described previously (Joosse *et al*, 2009; Bruin *et al*, 2010). The human 3600 BAC/PAC genomic clone set, covering the full genome at 1 Mb spacing used for the production of our arrays, was obtained from the Welcome Trust Sanger Institute (http://www.sanger.ac.uk/). Information on this clone set can be obtained at the BAC/PAC Resources Center Website (http://www.bacpac.chori.org/). The whole library was spotted in triplicate on every slide. To prevent slide batch spotting bias, samples were hybridised in random order (http://www.microarrays.nki.nl/).

### Statistical analysis

A propensity score using the baseline clinical data was calculated to compare the three patient groups (M0, PM, LM). The propensity score was taken as the probability of liver metastases given a patients' age, sex, T-stage, N-stage and primary location in a multinomial logistic regression model (D'Agostino Jr, 1998; Gleisner *et al*, 2008).

Patients missing baseline clinical data were imputed using median values. The association between genetic data (*BRAF*, *KRAS*, *PIK3CA* mutations, PI(3)K signalling pathway (mutation in at least one of the three genes) and CIMP) and the occurrence of liver metastases was determined and adjusted for quartiles of the propensity score using exact Cochran–Mantel–Haenszel tests. Fisher's exact and Kruskal–Wallis tests were used to determine the strength of associations between baseline clinical and pathological characteristics and the occurrence of liver metastases.

### 20q gain status

To determine whether a tumour had a gained 20q arm, we calculated average total copy numbers (TC) for 20q in each tumour using the average log 2 (AL2) of the BAC clones mapped to 20q (equation (1)). We assumed 70% tumour tissue in each sample, making this a conservative estimate of copy number. 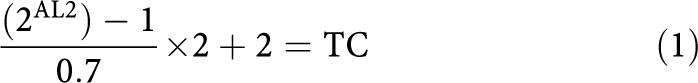


We labelled all tumours with a TC 20q of >2.5 as 20q amplified (Bruin *et al*, 2010).

## Results

### Patient characteristics

Table 1 presents the clinical and pathological characteristics for each of the three patient groups. Some of the patient characteristics are different among groups owing to the selection criteria employed for each group. These differences, however, were accounted for by using a propensity score for the evaluation of LM predictive markers (see below). More right-sided tumours were included in the M0 group, whereas the PM and LM group consisted of more left-sided colon and rectum tumours and of younger patients. There were more T4 tumours in the PM group compared with the M0 and LM group. As expected, the M0 group showed less N+ tumours. All patients in the M0, LM group and the metachrone patients in the PM group achieved an R0 resection for their primary tumour. The range of follow-up in the M0 group of patients still alive without recurrent disease was 4.6–16.7 years (median 8 years). Five patients in the M0 group died before 5 years follow-up without metastases. Range of time between primary diagnosis and diagnosis metastasis for metachrone LM and PM patients is 3.4–37.5 and 5.5–94.0 months, respectively.

### Mutation analysis

#### *KRAS*, *PIK3CA* and *BRAF* and PI(3)K signalling pathway mutations

In the combined study population (*n*=133), *BRAF* mutations were identified in 11 cases (8%), *KRAS* in 42 cases (32%) and *PIK3CA* in 21 cases (16%) (Table 2).

**Figure 1:**
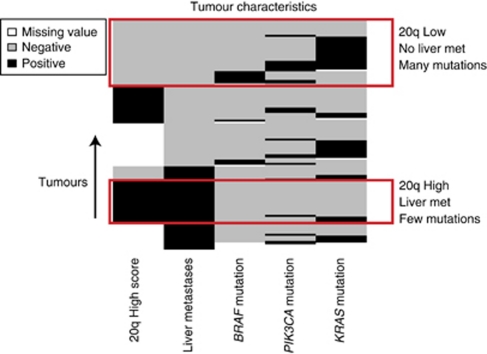
This figure shows the relation between the 20q gain status, liver metastasis (LM) and the mutation status of *BRAF*, *PIK3CA* and *KRAS*. Black means the patient is positive for either 20q gain, LM or one of the mutations, whereas gray means that the patient was negative for these measurements. White values indicate that these patients were not tested for the condition. Red boxes indicate two distinct patient population, highlighting the relation between 20q gain, LM and the mutations.

No significant differences were observed for the propensity score adjusted differences of *PIK3CA*, *KRAS* and *BRAF* mutations among the three groups (Table 2). *KRAS* and *BRAF* mutations were not observed in the same tumour, which is consistent with previous observations (Figure 1) (He *et al*, 2009).

For further analyses, we combined the M0 with the PM group because no statistical differences were found for mutations in the PI(3)K signalling pathway or the 20q status between these groups (*P*=0.83 and 0.86, respectively). The LM group showed a trend of fewer *KRAS* mutations compared with the M0–PM group (*P*=0.06) and no *BRAF* mutations (Table 2).

In total, 62 (47%) cases showed activation of the PI(3)K signalling pathway by having mutation in at least one of the three genes (Table 2), where 50 (38%) cases had mutations in a single gene and 12 (9%) cases had mutations in two genes (*PIK3CA* and *BRAF* or *PIK3CA* and *KRAS*). We note that two patients were missing either *KRAS* or *BRAF* data; however, they were classified as having a pathway mutation owing to harbouring a *PIK3CA* mutation. For nine patients, none of the mutations could be assessed (7%). Remarkably, mutation of the PI(3)K signalling pathway was significantly less present in the LM group compared with the combined M0–PM group (*P*=0.003). The LM group showed 27% (13 out of 48) PI(3)K signalling pathway mutations, whereas this was 62% (24 out of 39) and 54% (25 out of 46) in the M0 and PM group, respectively.

#### CpG-island methylator phenotype

CpG-island methylator phenotype status was significantly different among the three groups (*P*=0.03) (Table 2). The LM group revealed the lowest CIMP-high rate of 4% (2 out of 48), whereas the M0 and PM groups had 18 and 30% of CIMP-high, respectively. This remained significantly different when the LM group was compared with the M0–PM group (*P*=0.05). The difference between M0 and PM was not significant (*P*=0.18). According to the literature (Samowitz *et al*, 2005; Ogino *et al*, 2006), CIMP-high is tightly associated with *BRAF* mutation, which was also seen in our series (*P*=0.003) (Table 3).

#### Chromosomal instability and PI(3)K signalling pathway mutations

Our recent study had shown that the three groups had an overall similar pattern of chromosomal aberrations. Furthermore, chromosome 20q was significantly more gained (20q high; above median of the mean log 2 20q) in the LM group compared with the M0–PM group (KSE, *P*<0.05, Bonferroni multiple testing corrected) (Bruin *et al*, 2010).

Here we observed a strong inverse correlation between 20q amplification and PI(3)K signalling pathway mutation (*P*<0.0001) (Table 4a). Predominantly patients who were 20q low showed a PI(3)K signalling pathway mutation and *vice versa* (Table 4a and Figure 1). Table 4b shows that patients who developed LM were characterised by primary CRC with a 20q high (amplified) (62.5%) profile and a wild-type PI(3)K signalling pathway, whereas the M0–PM patients by a 20q low (49.1%) and a mutated PI(3)K signalling pathway (Table 4b). In the LM group, only 12.5% of the patients were 20q high in combination with a mutation in the PI(3)K signalling pathway; 9.4% showed 20q low with a mutation in the PI(3)K signalling pathway and 15.6% was 20q low with a wild-type PI(3)K signalling pathway. The overall comparison of the distributions of 20q and PI(3)K between LM and M0–PM patients was significant (*P*=0.0002).

#### MSI status

Four out of 133 patients were identified as MSI-high, with three MSI-high patients in the M0 group and one in the PM group. No association between MSI and CIMP-high was observed (Table 3).

## Discussion

The current standard for early detection of CRC liver metastases is the evaluation by imaging tools including ultrasound, MRI and PET/CT (Mauchley *et al*, 2005; Ruers *et al*, 2009). Furthermore, periodic clinical assessment and laboratory tests (e.g., carcinoembryonic-antigen tests, liver-function tests and complete blood counts) are performed to monitor signs of recurrence. However, these surveillance strategies have limitations owing to the lack of sufficient sensitivity and specificity (Pfister *et al*, 2004).

It is generally believed that circulating tumour cells, tumour-specific DNA methylation markers, gene expression profiles and MSI profiling may indicate which CRC patients are at higher risk to develop a recurrence. In addition, chromosomal instability of specific chromosomes, for example, 18q or 20q, have been associated with poor prognosis and related to liver metastases, respectively (Nanashima *et al*, 1997; Hidaka *et al*, 2000; Eschrich *et al*, 2005; Popat *et al*, 2005; Barrier *et al*, 2006; Cardoso *et al*, 2007; Lin *et al*, 2007; Anjomshoaa *et al*, 2008; Bruin *et al*, 2010; Kim *et al*, 2010; Salazar *et al*, 2011).

In this study of primary colorectal tumours, we aimed to evaluate the combined value of mutations in the PI(3)K signalling pathway, the CIMP, MSI and the aberration of chromosome 20q in primary colorectal tumours to predict the occurrence of liver metastases. We found that the primary tumours of patients who developed liver metastases are characterised not only by significantly higher amplifications of chromosome 20q (*P*=0.003), but also by significantly lower mutations in the PI(3)K signalling pathway (*P*=0.003) and hardly ever CIMP-high (*P*=0.05). We revealed a strong inverse association between 20q amplification and mutations in the PI(3)K pathway.

Chromosome 20q has previously been related to tumour progression, worse patient survival and observed in liver metastases (Aust *et al*, 2004; Bruin *et al*, 2010). Consequently, a further understanding of the candidate genes located on chromosome 20q amplification may guide us to understand the biological mechanisms in the development of liver metastases. Several genes located on 20q, for example, the oncogenes located on 20q13.1, such as *CAS/CSE1L*, *NABC1*, *ZNF217*, *Aurora2* (*BTAK*, *STK15*) and the ubiquitin-conjugating enzyme *E2C* (*UBE2C*), have been described to have an important role in tumour progression and liver metastases. Interestingly, none of these genes are related to the PI(3)K signalling pathway and therefore further strengthen the conclusion that these two phenomena are mutually exclusive (Aust *et al*, 2004; Rooney *et al*, 2004; Takahashi *et al*, 2006).

Several molecular studies have revealed that *KRAS* mutation status influences sensitivity to EGFR-targeted drugs (Normanno *et al*, 2009). Based on these findings, the European Medicines Agency and the US Food and Drug administration have placed restrictions on the usage of EGFR-targeted drugs and only approved for CRC metastatic patients with wild-type *KRAS* tumours. In patients without *KRAS* mutation, supplementary genotyping of *BRAF*, *NRAS* and *PIK3CA* could result in further improvement of response rates in the treatment with EGFR-targeted drugs. However, even a combination of all known mutations still leaves more than half of the non-responses unexplained (De Roock *et al*, 2010). This together with the results of our study may suggest that these patients do not depend on the PI(3)K signalling pathway and therefore might mean that EGFR-related therapy can be specially appropriate for patients with CRC liver metastasis.

Patients with LM selected for this study were treated with liver perfusion. These patients are characterised having multiple and/or in-operable metastases. This may influence the applicability of our conclusion for all CRC patients because it is unknown whether the biology of these patients is different from the overall group of patients who develop liver metastases. However, we have chosen this approach because these LM patients were representative for a group with high specificity for liver metastases development, that is, multiple metastases confined to the liver.

Despite the fact that patients in this study were selected and may not represent the general CRC patient population, our findings do reveal that patients at risk for developing liver metastases could be identified based on molecular characteristics of the primary tumour. Such patients should be frequently screened with modern imaging tools and are most likely to benefit from additional chemotherapy or targeted drugs. Further research needs to be carried out to validate these findings in an independent cohort of patients.

Molecular changes such as the presence of 20q amplification combined with the absence of mutations in the PI(3)K signalling pathway and low CIMP in a CRC primary tumour might become a reason to perform tailored follow-up and eventually targeted therapy.

## Figures and Tables

**Table 1:** Patient characteristics

	**M0 (*n*=39)**	**PM (*n*=46)**	**LM (*n*=48)**	**Total (*n*=133)**	***P*-value** a
*Gender*					0.001
Male	17 (44%)	23 (50%)	38 (79%)	78 (59%)	
Female	22 (56%)	23 (50%)	10 (21%)	55 (41%)	
					
*Age*					<0.0001
Median (range)	63 (37–86)	51 (30–72)	55 (36–69)	56 (30–86)	
					
*Location*					<0.0001
Right colon	19 (49%)	13 (28%)	3 (6%)	35 (26%)	
Left colon	14 (36%)	29 (63%)	32 (67%)	75 (56%)	
Rectum	6 (15%)	4 (9%)	13 (27%)	23 (17%)	
					
*T*					0.03
T2	0 (0%)	1 (2%)	5 (10%)	6 (5%)	
T3	33 (85%)	34 (74%)	38 (79%)	105 (79%)	
T4	6 (15%)	11 (24%)	3 (6%)	20 (15%)	
Missing	0 (0%)	0 (0%)	2 (4%)	2 (2%)	
					
*N*					0.001
N0	30 (77%)	19 (41%)	18 (38%)	67 (50%)	
N+	9 (23%)	26 (57%)	27 (56%)	62 (47%)	
Missing	0 (0%)	1 (2%)	3 (6%)	4 (3%)	
					
*Presentation*					0.004
Synchrone	0 (0%)	17 (37%)	33 (69%)	50 (38%)	
Metachrone	0 (0%)	29 (63%)	15 (31%)	48 (33%)	
Missing	39 (100%)	0 (0%)	0 (0%)	39 (29%)	
					
*Adjuvant chemotherapy*					<0.0001^b^
No	33 (85%)	10 (22%)	10 (21%)	53 (40%)	
Yes	4 (10%)	19 (41%)	5 (10%)	28 (21%)	
Synchrone^b^	0 (0%)	17 (37%)	33 (69%)	50 (38%)	
Missing	2 (5%)	0 (0%)	0 (0%)	2 (2%)	
					
*RT pretreatment*					0.004
Yes	0 (0%)	3 (7%)	9 (19%)	12 (9%)	
No	39 (100%)	43 (93%)	38 (79%)	120 (90%)	
Missing	0 (0%)	0 (0%)	1 (2%)	1 (<1%)	

Abbreviations: CRC=colorectal cancer; HIPEC=hyperthermal intraperitoneal chemotherapy; LM=liver metastasis; PM=peritoneal metastasis.

a *P*-value: association of clinicopathological characteristic with CRC subgroup.

b Patients who presented with synchronous metastasis in the LM and PM group could have received adjuvant chemotherapy as a part of their metastasis treatment (HIPEC or liver perfusion). Of note, this adjuvant treatment was given after metastasis treatment and therefore had no influence on the metastasis development.

**Table 2:** Molecular analysis of primary CRC of M0, PM and LM patients

					**M0 *vs* PM *vs* LM**	**LM *vs* M0-PM**
	**M0 (*n*=39)**	**PM (*n*=46)**	**LM (*n*=48)**	**Total (*n*=133)**	**CMH exact *P*-value** a	**CMH exact *P*-value** a
*BRAF*						
Wild type	33 (85%)	38 (83%)	44 (92%)	115 (86%)		
Mutated	5 (13%)	6 (13%)	0 (0%)	11 (8%)	0.23	0.18
Missing	1 (3%)	2 (4%)	4 (8%)	7 (5%)		
						
*KRAS*						
Wild type	23 (59%)	27 (59%)	33 (69%)	83 (62%)		
Mutated	16 (41%)	16 (35%)	10 (21%)	42 (32%)	0.12	0.06
Missing	0 (0%)	3 (7%)	5 (10%)	8 (6%)		
						
*PIK3CA*						
Wild type	30 (77%)	39 (85%)	40 (83%)	109 (82%)		
Mutated	9 (23%)	7 (15%)	5 (10%)	21 (16%)	0.2	0.25
Missing	0 (0%)	0 (0%)	3 (6%)	3 (2%)		
						
*PI(3)K signalling pathway* b						
Wild type	14 (36%)	17 (37%)	31 (65%)	62 (47%)		
Mutated	24 (62%)	25 (54%)	13 (27%)	62 (47%)	0.01	0.003
Missing	1 (3%)	4 (9%)	4 (8%)	9 (7%)		
						
*CIMP*						
Low	32 (82%)	32 (70%)	44 (92%)	108 (81%)		
High	7 (18%)	14 (30%)	2 (4%)	23 (17%)	0.03	0.05
Missing	0 (0%)	0 (0%)	2 (4%)	2 (2%)		
						
*20q Amplification* c						
Low	15 (38%)	23 (50%)	8 (17%)	46 (35%)		
High	10 (26%)	12 (26%)	25 (52%)	47 (35%)	0.01	0.003
Missing	14 (36%)	11 (24%)	15 (31%)	40 (30%)		

Abbreviations: BRAF=B-type Raf kinase; CIMP=CpG-island methylator phenotype; CRC=colorectal cancer; LM=liver metastasis; PI(3)K=phosphoinositide 3-kinase; *PIK3CA*=phosphoinositide-3-kinase, catalytic, alpha polypeptide; PM=peritoneal metastasis.

a CMH=Cochran–Mantel–Haenszel tests propensity score adjusted *P*-values.

b A patient with mutations in one or more of the three genes (*BRAF*, *KRAS*, *PIK3CA*) was counted as a mutated case in PI(3)K signalling pathway.

c 20q low amplifications are defined as amplifications smaller than the median of the mean 20q (cut-off 0.224 log 2) and 20q high *vice versa*.

**Table 3:** Co-occurrence in all primary CRC of CIMP, BRAF and MSI

	**CIMP-low (*n*=108)**	**CIMP-high (*n*=23)**	**Total (*n*=131)**	***P*-value** **Fisher's exact**
*BRAF*				
Wild type	98 (91%)	15 (65%)	113 (86%)	0.003
Mutated	5 (5%)	6 (26%)	11 (8%)	
Missing	5 (5%)	2 (9%)	7 (5%)	
				
*MSI*				
Stable/low	104 (96%)	17 (74%)	121 (92%)	0.11
High	2 (2%)	2 (9%)	4 (3%)	
Missing	2 (2%)	4 (17%)	6 (5%)	

Abbreviations: BRAF=B-type Raf kinase; CIMP=CpG-island methylator phenotype; CRC=colorectal cancer; MSI=microsatellite instability.

**Table 4a:** Co-occurrence in all primary CRC of PI(3)K signalling pathway mutation and chromosomal amplification of 20q

	**20q Low**	**20q High**	**Missing**	**All**	***P*-value Fisher's exact**
No PI(3)K pathway mutation	14	33	15	62	<0.0001
PI(3)K Pathway mutation	31	11	20	62	
Missing	1	3	5	9	
All	46	47	40	133	

Abbreviations: CRC=colorectal cancer; PI(3)K=phosphoinositide 3-kinase.

**Table 4b:** Co-occurrence in primary CRC of PI(3)K signalling pathway mutation and chromosomal amplification of 20q for M0+PM vs LM patients

		**M0+PM**		**LM**	
**20q**	**PI(3)K pathway**	***n***	**%**	***n***	**%**
Low	Wild type	9	15.8	5	15.6
	Mutated	28	49.1	3	9.4
High	Wild type	13	22.8	20	62.5
	Mutated	7	12.3	4	12.5

Abbreviations: CRC=colorectal cancer; LM=liver metastasis; PI(3)K=phosphoinositide 3-kinase; PM=peritoneal metastasis.
